# Acceptance of Booster COVID-19 Vaccine and Its Association with Components of Vaccination Readiness in the General Population: A Cross-Sectional Survey for Starting Booster Dose in Japan

**DOI:** 10.3390/vaccines10071102

**Published:** 2022-07-08

**Authors:** Mikiko Tokiya, Megumi Hara, Akiko Matsumoto, Mohammad Said Ashenagar, Takashi Nakano, Yoshio Hirota

**Affiliations:** 1 Department of Social and Environmental Medicine, Faculty of Medicine, Saga University, 5-1-1 Nabeshima, Saga 849-8501, Japan; matsumoa@cc.saga-u.ac.jp (A.M.); sx5080@cc.saga-u.ac.jp (M.S.A.); 2 Department of Preventive Medicine, Faculty of Medicine, Saga University, 5-1-1 Nabeshima, Saga 849-8501, Japan; harameg@cc.saga-u.ac.jp; 3 Department of Pediatrics, Kawasaki Medical School, 577 Matsushima, Kurashiki, Okayama 701-0192, Japan; ndhkk029@ybb.ne.jp; 4 Clinical Epidemiology Research Center, SOUSEIKAI Medical Group (Medical Co. LTA), 3-5-1 Kashiiteriha, Higashi-ku, Fukuoka 813-0017, Japan; hiro8yoshi@lta-med.com

**Keywords:** vaccine, readiness, scale, intention, 7C, social norms, survey, epidemiology

## Abstract

The Japanese government approved COVID-19 vaccine booster doses in November 2021. However, intentions and readiness for booster vaccines among the general population were unknown. This survey measured the intentions for COVID-19 booster vaccination. Among 6172 participants (53.2% female), 4832 (78.3%) accepted booster doses; 415 (6.7%) hesitated. Vaccination intention was associated with higher age, marital status, having children, underlying diseases, and social norms. To evaluate the readiness for vaccination, the seven component (7C) vaccination readiness scale was employed, comprising “Confidence”, “Complacency”, “Constraints”, “Calculation”, “Collective responsibility”, “Compliance”, and “Conspiracy”. Participants with acceptance showed significantly higher 7C scores (*p* < 0.001) than those who hesitated or were unsure. Multivariable logistic regression analysis revealed that the “social norms” predictor was the strongest predictor of acceptance (adjusted odds ratio (AOR) 4.02, 95% confidence interval (CI): 3.64–4.45). “Constraints” (AOR: 2.27, 95% CI: 2.11–2.45) and “complacency” (AOR: 2.18, 95% CI: 2.03–2.34) were also strongly associated with acceptance, but “compliance” (AOR: 1.24, 95% CI: 1.18–1.31) and “conspiracy” (AOR: 1.42, 95% CI: 1.33–1.52) were weakly associated. The “7C vaccination readiness scale” is useful for measuring vaccine acceptance in the Japanese population. However, “social norms” might be more suitable than “compliance” and “conspiracy” for measuring vaccine acceptance in Japan.

## 1. Introduction

The coronavirus disease 2019 (COVID-19) pandemic has not yet ended, and mutant strains continue to appear. In Japan, the COVID-19 vaccine has been provided free of charge to all residents since February 2021, and, as of 1 December 2021, approximately 80% of the population aged ≥12 years had received two doses [1]. However, the effectiveness of the vaccines in “preventing infection”, “preventing disease onset”, and “preventing severe disease” gradually declined [2]. Therefore, to prevent the spread of infection and severe disease, the Japanese government planned a booster vaccination between 1 December 2021 and 30 September 2022 [3]. Since controlling the COVID-19 pandemic requires higher immunity among the population, a higher acceptance rate of the booster vaccine is needed. Our previous studies have shown that the acceptance of vaccination against COVID-19 had temporarily decreased in the initial stages of the vaccine program; however, it increased in the later stages [4]. In addition, our study showed that vaccine confidence can possibly lead to vaccine acceptance [4]. Furthermore, Halbrook et al. [5] also reported that confidence in the COVID-19 vaccine and vaccination proportion have increased over time in the United States [5]. Several groups have investigated the intention to be vaccinated with the booster dose among the general adult population, and the results varied across regions. In Italy, a random sampling survey was conducted 2 weeks before the booster dose became available, and the acceptance of the booster dose was found to be 85.7% [6]; the main reasons for the willingness of the participants to receive the dose were so that they would protect themselves and their relatives [6]. In the United States, a web survey conducted 2 months before the booster dose became available showed a 79.1% acceptance rate [7], and a significantly higher proportion of booster dose-hesitant individuals had very little to no trust in the COVID-19 vaccine information provided by public health/government agencies compared with non-hesitant individuals [7]. In Poland, a web survey conducted approximately 3 months before the booster dose became available showed a 71.0% acceptance rate [8], and the main reasons against accepting a booster COVID-19 dose included the side effects experienced following previous doses, the opinion that further vaccination is unnecessary, and safety uncertainties [8]. Therefore, the acceptance of booster vaccination may change temporally during the initiation of the booster vaccination. There are few reports on the intentions of receiving the booster dose vaccination in Japan. Sugawara et al. [9] surveyed medical students approximately 6 months before the booster dose and reported a high booster dose readiness (84.5%) [9], and willingness to receive the third dose was associated with confidence in the protection offered by a COVID-19 vaccination [9]. In contrast, a web-based survey conducted approximately 2 months before the booster dose for the general adult population reported the intention of receiving booster vaccination to be 47.4% [10]; however, the factors associated with the willingness for booster vaccination are unclear. The lower acceptance among Japanese adults might result in a lower vaccination rate for the booster dose. The booster dose administration at approximately 3 months after the start of vaccinations in Japan was approximately 16.5%, but the booster uptake has subsequently slowed down [11]. To increase the acceptance rate of booster doses among Japanese adults, it is necessary to examine the potential factors that may act as barriers.

Social norms are reported to be a key driver of behavior in the COVID-19 pandemic [12,13]. Japanese people, in general, are likely to decide by observing the people around them. We determined that social norm was associated with acceptance of the priming vaccination (Table S1) [4,14]. COVID-19 vaccination willingness was reportedly associated with the high espousal of social norms among patients from one Japanese primary care clinic [15]. However, whether social norms influence the intention to receive booster vaccines among the general population is unknown.

It has been reported that vaccination coverage depends on vaccine availability and the public’s readiness to be vaccinated [16]. Regarding vaccination readiness, five components (5C), such as confidence, complacency, constraints, calculation, and collective responsibility, have been reported to influence the acceptance rate of several vaccines [17]. Recently, Geiger et al. [16] proposed two additional components of vaccine readiness [16]. The first one is compliance; a mandatory immunization policy by the government affects the public’s psychological perception and decreases their intention to be immunized. This accounts for the importance of compliance as a factor. The second one is conspiracy, which has been associated with lower vaccination intentions and lower support for government regulation. It is unknown whether these seven components (7Cs) can serve as predictors of vaccination intention in the Japanese population, particularly their intent to receive a third vaccination (booster dose).

This study aimed to investigate the intention to receive a booster dose among Japanese adults and to clarify the factors associated with acceptance or barriers to vaccination using the 7C scale to contribute to vaccination policy.

## 2. Materials and Methods

### 2.1. Survey Participants and Data Collection

Between 20 December 2021 and 22 December 2021, a web-based, cross-sectional survey was conducted by a web-survey company (Macromill, Inc., Tokyo, Japan), which registered approximately 1.2 million people in the research panel, to measure the intention of receiving the booster dose and its associated factors among the general Japanese population using the Japanese version of the “7C vaccination readiness scale” [16]. Our previous studies reported the survey details [4,14]. The calculated sample size was between 2000 and 5000, considering the following: α = 0.05/number of survey items (50 items) = 0.001, β = 0.20, odds ratio: 1.5, 30–50% hesitancy to vaccinate, and 10–20% possession of relevant factors. We continued recruitment until 7000 applicants were included. We sent an e-mail to men and women between the age of 20 and 79 years with the help of an internet research firm, requesting their cooperation in the survey. Participation was voluntary, and points, which could be used to buy products and services from corporate sponsors, were given to those who participated in the survey. We clearly stated that “the questionnaire is a part of our research and may be published after statistical processing while maintaining anonymity”, and informed consent was obtained from the participants. We placed the questionnaire in a secure section of our website. After the participants responded, we verified that no variables were missing. We obtained approval from the Ethics Committee of Saga University (No: R2-24) to conduct this study.

### 2.2. Measurement Method

#### 2.2.1. Sociodemographic Factors, COVID-19 Incidence, and Adverse Events in Priming

Sociodemographic factors included sex, age group (20–29, 30–39, 40–49, 50–59, 60–69, 70–79 years), occupation, area of residence, marital status (married, unmarried), presence of children, annual household income category (below or above JPY 4 million), highest educational qualification (high school, vocational school, or university), height and weight, underlying diseases, and smoking status. Participants were also asked whether they or their close relatives had ever been infected with COVID-19, whether they had a history of close contact, and whether they had undergone polymerase chain reaction or antigen testing for detecting COVID-19. We asked participants who had received the priming vaccine about adverse events (AEs), including lumps, itching, pain, redness, swelling, fever, tiredness, fatigue, headache, chills, vomiting, diarrhea, muscular pain, arthralgia, and anaphylactic shock.

#### 2.2.2. COVID-19 Vaccine History and Intention to Be Vaccinated with a Booster Dose

Participants were asked about the number of doses of the COVID-19 vaccination that they had received. The respondents who had completed two doses of the COVID-19 vaccine were asked to select the most suitable view on “I would like to get the third vaccination (booster) when I get the information”, on a 5-point scale, where “Strongly disagree”, “Disagree”, “Neither or not”, “Agree”, and “Strongly Agree” corresponded to a score of 1, 2, 3, 4, and 5, respectively.

#### 2.2.3. 7C Vaccination Readiness Scale

We used the Japanese version of the vaccination readiness scale (7Cs) [16,18,19], which contains the following seven statements: Q1: “I am convinced the appropriate authorities do only allow effective and safe vaccines (Confidence);” Q2: “I get vaccinated because it is too risky to get infected (Complacency);” Q3: “Vaccinations are so important to me that I prioritize getting vaccinated over other things (Constraints);” Q4: “I only get vaccinated when the benefits outweigh the risks (Calculation);” Q5: “I see vaccination as a collective task against the spread of diseases (Collective Responsibility);” Q6: “It should be possible to sanction people who do not follow the vaccination recommendations by health authorities (Compliance);” Q7: “Vaccinations cause diseases and allergies that are more serious than the diseases they ought to protect from (Conspiracy)”. The scores for “Confidence”, “Complacency”, “Constraints”, “Collective Responsibility”, and “Compliance” were set as follows: “Strongly disagree” = 1 point, “Almost disagree” = 2 points, “Probably disagree” = 3 points, “Neither or not” = 4 points, “Probably agree” = 5 points, “Almost agree” = 6 points, and “Strongly agree” = 7 points. The scoring for “Calculation” and “Conspiracy” was the reverse of the above. The higher the score was, the higher the vaccination readiness was [16,18,19]. The Cronbach’s alpha was 0.66 for 5C of 7C and 0.64 for 7C.

#### 2.2.4. Vaccine Social Norms

Participants were asked to respond to “If most people take a booster dose, I will do too” (Social norms). We obtained responses on a 5-point scale, as above.

The Cronbach’s alpha between “I would like to be vaccinated if the vaccine is approved” and “I would like to be vaccinated if everyone else is vaccinated” in a previous survey (*n* = 7210) [4,14] (data not publicly available) was 0.85.

### 2.3. Statistical Analyses

We conducted a cross-sectional analysis. All tests were performed using categorical variables. For the intention to receive the booster dose, those who had already received two vaccine doses and had not received the third dose were included in the analysis. Among 7210 participants, 848 had never been vaccinated, 69 had been vaccinated with one dose, 6172 had received two doses, and 121 had received three doses. Thus, in this study, we analyzed 6172 participants.

We categorized the intention to be vaccinated into the following three groups: “strongly disagree” and “disagree” as “hesitancy”, “neither or not” as “not sure”, and “agree” and “strongly agree” as “acceptance”.

Attributes and other comparisons were made by vaccination intention as described above, and the χ^2^ test was performed to evaluate the differences. Similarly, a χ^2^ test was performed for the presence of adverse events since the last vaccination based on the intention to receive the COVID-19 booster vaccine. Next, mean scores (7C) on the vaccination readiness scale were calculated as per vaccination intention and compared among the three groups using the Kruskal–Wallis test. Finally, logistic regression analysis was performed to examine the association between “acceptance” and attributes or 7C or “Social norms”, with a univariate analysis for Model 1 and adjustment for sex, age, children, underlying disease status, household income, and AEs of previous vaccination (fever, tiredness, fatigue, headache, chills, vomiting, diarrhea, muscular pain, arthralgia, and anaphylactic shock) for Model 2. However, sex, age, children, underlying disease status, and household income were adjusted all at once. Effect sizes (eta squared) were calculated using the General Linear Model adjusted for the variables in Model 2.

The significance level was set at a two-sided *p* < 0.001 using Bonferroni correction (0.05/50 items = 0.001). SAS version 9.4 (SAS Institute Inc., Cary, NC, USA) was used for statistical analyses.

## 3. Results

Table 1 shows the intention of the respondents to be vaccinated. Of the respondents, 2515 agreed to be vaccinated, 2317 strongly agreed, 925 did not know, 289 did not agree, and 126 strongly disagreed about getting vaccinated. The respondents were grouped as follows: 4832 (78.3%) as “acceptance”, 925 (15.0%) as “not sure”, and 415 (6.7%) as “hesitancy”.

Table 2 compares attributes and other information according to the intention to be vaccinated. Significant differences were found in sex, age, marital status/children, annual household income, presence of underlying disease, and “Social norms”.

Table 3 shows the number and percentage of study participants who experienced an AE following the priming vaccination based on the intention to receive the COVID-19 vaccine booster. The proportion of those who experienced fever, tiredness, fatigue, headache, cold, vomiting, diarrhea, muscular pain, and arthralgia as AEs was significantly higher among participants who were hesitant or not sure about the booster dose than among those whose who accepted the booster dose. Significant associations with “hesitation” were observed for vomiting (adjusted odds ratio (AOR): 1.80, 95% confidence interval (CI): 1.14–2.86), arthralgia (AOR: 1.64, 95% CI: 1.33–2.03), and fever (95% CI: 1.28, 95% CI: 1.10–1.49) (Table S2).

**Figure 1:**
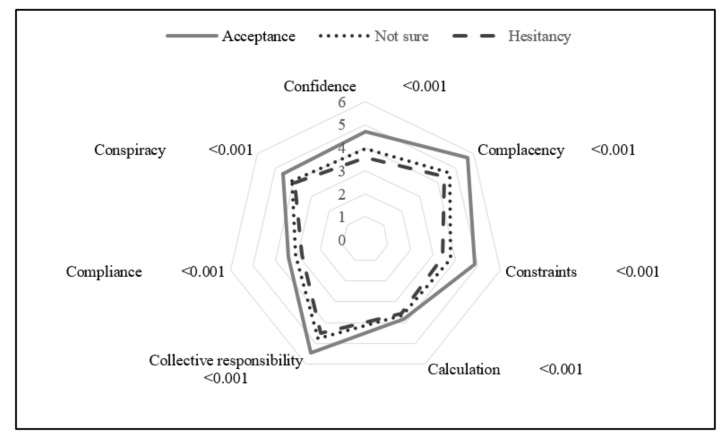
The 7C scale of vaccination readiness means’ score (age, sex-adjusted) according to the intention to be vaccinated (*p*-value calculated by Kruskal–Wallis test for “hesitancy”, “not sure”, and “acceptance” scores).

Figure 1 shows vaccination intention’s sex- and age-adjusted mean scores for 7C. “Acceptance” scoring was significantly higher than “not sure” or “hesitancy” (*p* < 0.001). In particular, the mean scores for “Complacency”, “Constraints”, and “Collective responsibility” were higher for “acceptance” than for the other groups. Considering “Confidence”, “Calculation”, “Compliance”, and” Conspiracy” among the 7Cs, the most frequent answer was “Neither or not” (Table S3).

Table 4 shows the association between “acceptance” and “7C” among vaccination intenders based on the logistic regression analysis. Even with adjustment factors, including AEs, the 7C scores were significantly associated with “acceptance”. However, the odds ratios for “Calculation”, Compliance”, and “Conspiracy” were not as high. In contrast, the AOR for “Social norms” was the highest, with the effect sizes also being larger (eta = 0.27).

## 4. Discussion

In this study, we investigated the intention to receive a booster vaccination and further examined the factors associated with vaccination acceptance and barriers using the 7C vaccination readiness scale.

### 4.1. Booster Dose Vaccination Intention

The booster dose was added to the primary two-dose vaccination, which was the initial immunization plan, by the government. This survey was conducted just before the start of the third vaccination (booster dose) for the general adult population. We expected a low percentage of acceptance for the booster dose. However, 78.3% of participants with two doses were classified into the “acceptance” category. This proportion was comparable to that of the United States study [7] and was more than the 71% reported in the Polish survey [8]. In contrast, this was lower than the 85.7% in the Italian study [6]. Because the approved COVID-19 vaccine is reported to be “highly effective in preventing severe disease and death” [20], we suspected that if the cumulative numbers of infections and deaths were high [21] and vaccination was mandatory [22], the proportion of acceptance would increase (Table S4). However, despite the smaller numbers of cumulative infections and deaths in Japan, the booster dose vaccination coverage and acceptance rate were higher than those in other countries.

In this study, AEs were associated with “hesitancy” and “not sure” for booster vaccination (Table 3 and Table S2). This association was also reported in a Polish study [8]. Since AEs may have a considerable impact on the acceptance of the booster dose, we adjusted for this to evaluate the associations between booster dose acceptance and 7C. The factor of “social norms” was the most significant factor that was associated with the acceptance of booster doses in this study. The AOR against the booster dose for “social norms” was 4.02 (95% CI: 3.64–4.45). The AOR against a priming vaccination (AOR: 8.02, 95% CI: 7.13–9.03) in a previous study was higher than the AOR against the booster dose (Table S1) [4,14]. The reason for this finding could be that the COVID-19 vaccination was a new experience for people. It has also been reported that normative beliefs, including social norms, reliably predict various health behaviors, including vaccination and physical activity [23]; we believe our results are consistent with previous research. Thus, our study supported social norms to be an essential driver of vaccination behavior [12].

### 4.2. Suitability of 7C Vaccination Readiness Scale for General Japanese Population

Mean scores of the 7Cs were higher in participants with “acceptance” than in those belonging to the “hesitancy” or “not sure” groups (Figure 1 and Figure S1). Even after adjusting for confounding factors, including AEs and “social norms”, the 7C had positive odds ratios (Table 4 and Table S2); the AORs for “Confidence”, “Complacence”, “Constraint”, and “Collective responsibility” were 1.76–2.27, while “Compliance” and “Conspiracy”, which are additional components recently introduced after COVID-19 [16,24,25], showed low odds ratios of 1.24–1.42. The low odds ratios for these two items may be because the participants of this study belonged to the general Japanese population. While vaccination is mandatory in many countries [16,25], in Japan, in contrast, vaccination is voluntary. Furthermore, the cumulative morbidity rate is relatively low, and behavioral restrictions are limited [21]. In the case of the influenza vaccine, a qualitative analysis of the beliefs of the authors of a Japanese anti-vaccine website revealed that they were not believers in conspiracy theories but that their anti-vaccine attitudes were motivated by two primary concerns: people’s “safety” and “self-esteem” [26]. This background peculiarity may be related to the low odds ratio for “Compliance”. Moreover, according to Hornsey et al. [24], the effect sizes of “Conspiracy” on acceptance were significant in countries where people have “the psychological roots of anti-vaccination attitudes”. However, in the Japanese population, the effect size of “Conspiracy” on acceptance was relatively small [24]. Therefore, we believe that 5C, which does not include “Compliance” and “Conspiracy”, may be sufficient to measure vaccination readiness in the Japanese population.

### 4.3. COVID-19 Vaccination Issue in the General Population

Acceptability was strongly associated with the “social norms” score in our study (AOR: 4.02, 95% CI: 3.64–4.45). This may result in an accelerated increase in acceptability under conditions of high vaccination rates and a delayed increase under conditions of low vaccination rates. Social norms are not a trend unique to Japan; a survey of college students in the United States reported the same phenomena [13]. The COVID-19 vaccine is a voluntary vaccination in Japan; the Ministry of Health, Labour and Welfare recommends that people weigh the benefits and risks and decide for themselves whether to be vaccinated [27]. This study suggests that there is no tendency for people to decide whether to be vaccinated based on individual judgment. It may be necessary to provide the information required by the general population to make a well-informed decision or, perhaps, social norm-based intervention strategies should be adopted.

Previous studies have shown that those who initially responded with “hesitancy” or “not sure” may later change their response to “acceptance” [4]. To change people’s vaccination intentions from “hesitancy” and “not sure” to “acceptance”, we believe it is effective to increase the scores of “Constraints”, “Complacency”, and “Confidence”, the three scale items that showed high AORs among the participants of this survey. As stated in the “5C Psychological Antecedents of Vaccination”, people with more “Constraints” feel that they do not have enough time for vaccination [17]. To increase the “Constraints” score, structural and psychological barriers need to be lowered, such as shorter travel distances and travel time for vaccinations [17,28]. It is also necessary to lean on the cause of the inconvenience of vaccinations. To increase the “Complacency” score, people should be informed about the risk of infecting others during incubation or the symptom-free period after infection. In the case of the Omicron strain, there is a reported risk of infected individuals spreading the virus to others, even in the absence of any symptoms. Moreover, the disease has been reported to vary in severity depending on the age and presence of co-morbidities in the infected person [29]. Therefore, it is necessary to take measures against the infection, even when people are symptom-free. To increase “Confidence”, it is essential for government agencies to gain reliability [30,31,32,33]. Confidence in medical care and information reliability have also influenced COVID-19 vaccination intentions [34]. A systematic review of vaccination intentions for the COVID-19 vaccine revealed the adverse effects of social media misinformation [35]. The need to provide residents with reliable information from local health care providers about the risks of disease and the benefit of vaccination has to be emphasized [35].

Thus, it is necessary to promote higher scores for “Calculation” in addition to “Constraint”, “Complacency”, “Confidence”, and “Collective responsibility” to increase the “acceptability” of COVID-19 vaccination among the Japanese population. The 7C can also be used as a readiness scale for the COVID-19 vaccine and other vaccinations [16]. In the future, it is necessary to verify the vaccination status of the Japanese population using the 7C scale against various vaccine targets.

### 4.4. Strengths and Limitations

The strength of this study is that the intention of the general Japanese population to vaccinate with the booster dose was surveyed just before the initiation of the booster dose phase of the COVID-19 vaccine program. We also measured the readiness of the third vaccination using the “7C vaccination readiness scale” published last year to identify issues in Japan. This is the first study to assess vaccination intentions using the 7C vaccination readiness scale and social norms regarding indicators and intention to be vaccinated.

However, this study had several limitations. First, we did not evaluate the level of knowledge concerning COVID-19, which could have impacted vaccine acceptance [36] in this study. Thus, it is necessary to evaluate this in future studies. Second, not all the target ages for booster doses were included in the survey because the research panel had limited participants, i.e., 18–19-year-olds (1.8% of the total population) and 80-year-olds or above (9.5% of the total population). Third, since the survey was web-based, the survey respondents could have been people who had easy access to the Internet and had better awareness about vaccination through the Internet. However, the distributions of age (generation) and area of residence of the participants were similar to those of Japan. The age groups of the participants were 10.8% in their 20s, 14.2% in their 30s, 17.1% in their 40s, 19.8% in their 50s, 18.7% in their 60s, and 19.4% in their 70s; as of October 2021, the Japanese population was, in descending order, 10.1%, 11.1%, 14.3%, 13.6%, 12.2%, and 13.1% in their 20s, respectively, which did not differ significantly from the proportions of the participants of this study (2 × 6 χ-square test χ^2^ = 0.65, *p* = 0.99). Furthermore, similar to age, no difference in the proportions was observed when the region of residence was compared with the 2015 data (2 × 8 χ-square test χ^2^ = 1.89, *p* = 0.97). To address the selection bias, we adjusted for the covariates using a multivariate logistic regression analysis; however, there are limitations in generalizing the findings to the overall Japanese population. Moreover, selection bias cannot be removed because the people registered with the survey company from which the participants were drawn only included those with access to the Internet and because points for purchases were awarded for participation. However, most surveys on the intention to receive the COVID-19 vaccine are conducted similarly, making comparisons with other surveys easier. Fourth, this cross-sectional study cannot refer to causal relationships. Follow-up is needed to determine if participants who belonged to the “acceptance” group have been vaccinated. Lastly, few studies use the “7C scale of vaccination readiness”. The impact of various targeted vaccines and different nationalities on the validity of the 7C scale needs further evaluation.

## 5. Conclusions

The general Japanese population was surveyed about their intention to receive the third dose of the COVID-19 vaccine and rated on the “7C vaccination readiness scale”. We found that the “7C vaccination readiness scale” is useful to measure vaccine acceptance in the Japanese population. However, “Compliance” and “Conspiracy” were not suitable for measuring vaccine acceptance. Based on our results, we believe that the indicator “social norms” could be a more suitable predictor of vaccine acceptance for the Japanese population. Public health measures for increasing scores of “Confidence”, “Complacency”, and “Constraints”, as well as increasing “social norms”, are necessary for the acceptance of booster vaccination in Japan.

## Figures and Tables

**Table 1:** Participant responses to “I would like to get the third vaccination (booster) when I get the information”.

	*n*	(%)
Strongly agree	2317	37.5
Agree	2515	40.8
Neither or not	925	15.0
Disagree	289	4.7
Strongly disagree	126	2.0

**Table 2:** Characteristics of participants with the intention to receive the booster dose of the COVID-19 vaccine.

		*n*	Acceptance *n* = 4832 *n* (%)	Not Sure *n* = 925 *n* (%)	Hesitancy *n* = 415 *n* (%)	*p*-Value
Sex	Male	2890	2319 (80.2)	386 (13.4)	185 (6.4)	0.001
	Female	3282	2513 (76.6)	539 (16.4)	230 (7.0)	
Age	(Mean: years old)		52.5	43.2	42.6	<0.001
	20–29	791	521 (65.9)	180 (22.8)	90 (11.4)	<0.001
	30–39	1054	688 (65.3)	254 (24.1)	112 (10.6)	
	40–49	1133	828 (73.1)	211 (18.6)	94 (8.3)	
	50–59	1151	957 (83.2)	137 (11.9)	57 (5.0)	
	60–69	1018	903 (88.7)	81 (8.0)	34 (3.3)	
	70–79	1025	935 (91.2)	62 (6.1)	28 (2.7)	
Area	Hokkaido	291	217 (74.6)	56 (19.2)	18 (6.2)	0.193
	Tohoku	336	266 (79.2)	40 (11.9)	30 (8.9)	
	Kanto	2373	1890 (79.7)	334 (14.1)	149 (6.3)	
	Chubu	994	755 (76.0)	164 (16.5)	75 (7.6)	
	Kinki	1211	945 (78.0)	182 (15.0)	84 (6.9)	
	Chugoku	307	232 (75.6)	56 (18.2)	19 (6.2)	
	Shikoku	145	117 (80.7)	20 (13.8)	8 (5.5)	
	Kyusyu	515	410 (79.6)	73 (14.2)	32 (6.2)	
Married	No	2275	1680 (73.9)	402 (17.7)	193 (8.5)	<0.001
	Yes	3897	3152 (80.9)	523 (13.4)	222 (5.7)	
Child	No	2507	1822 (72.7)	470 (18.8)	215 (8.6)	<0.0001
	Yes	3665	3010 (82.1)	455 (12.4)	200 (5.5)	
Annual household income	<JPY 4 million	1654	1312 (79.3)	234 (14.2)	108 (6.5)	<0.001
	≥JPY 4 million	3170	2539 (80.1)	440 (13.9)	191 (6.0)	
	Unknown	1348	981 (72.8)	251 (18.6)	116 (8.6)	
Educational attainment	High school graduate	1781	1404 (78.8)	251 (14.1)	126 (7.1)	0.389
	Above higher education	4391	3428 (78.1)	674 (15.4)	289 (6.6)	
Body mass index ≥ 25 kg/m^2^	No	5013	3885 (77.5)	773 (15.4)	355 (7.1)	0.005
	Yes	1159	947 (81.7)	152 (13.1)	60 (5.2)	
Underlying disease	No	3965	2956 (74.6)	698 (17.6)	311 (7.8)	<0.001
	Yes	2207	1876 (85.0)	227 (10.3)	104 (4.7)	
Smoking	No	5225	4075 (78.0)	787 (15.1)	363 (7.0)	0.218
	Yes	947	757 (79.9)	138 (14.6)	52 (5.5)	
SARS-CoV-2 infection status (myself)	No	6116	4790 (78.3)	916 (15.0)	410 (6.7)	0.870
	Yes	56	42 (75.0)	9 (16.1)	5 (8.9)	
SARS-CoV-2 infection status (family, etc.)	No	5396	4241 (78.6)	811 (15.0)	344 (6.4)	0.016
	Yes	776	591 (76.2)	114 (14.7)	71 (9.2)	
SARS-CoV-2 infection status (inspection only)	No	5344	4191 (78.4)	807 (15.1)	346 (6.5)	0.127
	Yes	828	641 (77.4)	118 (14.3)	69 (8.3)	
If most people take a booster dose, I will do too (Social norms)	Strongly disagree	151	67 (44.4)	4 (2.7)	80 (53.0)	<0.001
	Disagree	326	133 (40.8)	26 (8.0)	167 (51.2)	
	Neither or not	1159	481 (41.5)	560 (48.3)	118 (10.2)	
	Agree	2766	2401 (86.8)	319 (11.5)	46 (1.7)	
	Strongly agree	1770	1750 (98.9)	16 (0.9)	4 (0.2)	

*p*-values were calculated using the 3 × *n* χ^2^ test. Mean age was calculated using a one-way analysis of variance. The Bonferroni method defined a significance level of less than 0.001 as 0.05/50 = 0.001. SARS-CoV-2 infection status (family, etc.) refers to family members, friends, and business associates.

**Table 3:** Adverse events of previous vaccination by intention to receive the booster dose of the COVID-19 vaccine.

Adverse Events/Yes	Acceptance *n* = 4832 *n* (%)	Not Sure *n* = 925 *n* (%)	Hesitancy *n* = 415 *n* (%)	*p-*Value *
Lumps at the vaccination site	870 (18.0)	155 (16.8)	58 (14.0)	0.093
Itching at the vaccination site	626 (13.0)	144 (15.6)	64 (15.4)	0.052
Pain at the vaccination site	3210 (66.4)	632 (68.3)	275 (66.3)	0.525
Redness at the vaccination site	979 (20.3)	198 (21.4)	79 (19.0)	0.577
Swelling of the inoculation site	1517 (31.4)	299 (32.3)	152 (36.6)	0.086
Fever	2142 (44.3)	517 (55.9)	259 (62.4)	<0.001
Tiredness, fatigue	2039 (42.2)	491 (53.1)	234 (56.4)	<0.001
Headache	1036 (21.4)	293 (31.7)	157 (37.8)	<0.001
Chills	534 (11.1)	167 (18.1)	97 (23.4)	<0.001
Vomiting	64 (1.3)	24 (2.6)	17 (4.1)	<0.001
Diarrhea	80 (1.7)	19 (2.1)	24 (5.8)	<0.001
Muscular pain	1092 (22.6)	266 (28.8)	149 (35.9)	<0.001
Arthralgia	450 (9.3)	148 (16.0)	87 (21.0)	<0.001
Anaphylactic shock	11 (0.2)	3 (0.3)	5 (1.2)	0.003
None	457 (9.5)	76 (8.2)	30 (7.2)	0.186

* *p*-values were calculated using the 3 × 2 χ^2^ test.

**Table 4:** Logistic regression analysis of factors associated with acceptance of the booster dose of vaccination.

	Crude OR	95% CI	*p-*Value	Adjusted * OR	95% CI	*p-*Value	Eta **
Sex	0.81	0.71–0.91	0.001	0.95	0.81–1.10	0.483	0.07
Age	1.46	1.40–1.52	<0.001	1.39	1.31–1.47	<0.001	
Child	1.73	1.53–1.95	<0.001	0.98	0.83–1.15	0.777	
Annual household income	1.05	0.91–1.22	0.526	1.27	1.08–1.49	0.003	
Underlying disease	1.94	1.69–2.22	<0.001	1.30	1.10–1.54	0.003	
Social norms	3.98	3.66–4.34	<0.001	4.02	3.64–4.45	<0.001	0.27
Confidence	1.80	1.71–1.90	<0.001	1.76	1.65–1.87	<0.001	0.13
Complacency	2.34	2.20–2.49	<0.001	2.18	2.03–2.34	<0.001	0.18
Constraints	2.41	2.26–2.56	<0.001	2.27	2.11–2.45	<0.001	0.18
Calculation	1.12	1.07–1.17	<0.001	1.10	1.04–1.16	0.001	0.07
Collective responsibility	1.83	1.73–1.93	<0.001	1.77	1.65–1.90	<0.001	0.13
Compliance	1.25	1.19–1.31	<0.001	1.24	1.18–1.31	<0.001	0.08
Conspiracy	1.53	1.45–1.62	<0.001	1.42	1.33–1.52	<0.001	0.09

* Adjusted for sex, age, children, underlying disease status, household income, and adverse events of previous vaccination (fever, tiredness, fatigue, headache, cold, vomiting, diarrhea, muscular pain, arthralgia, and anaphylactic shock). However, sex, age, children, underlying disease status, and household income were adjusted all at once. ** The effect size (eta squared) was calculated using the General Linear Model, adjusted for age, children, underlying disease status, household income, and adverse events of previous vaccination (fever, washed-out feeling, headache, cold, vomiting, diarrhea, muscular pain, arthralgia, anaphylactic shock). OR: odds ratio; CI: confidence interval.

## Data Availability

The data presented in this study are available on request from the corresponding author (M.T.). The data are not publicly available due to privacy concerns.

## Supplementary Materials

The following are available online at https://www.mdpi.com/article/10.3390/vaccines10071102/s1. Table S1: Logistic regression analysis of factors associated with acceptance of the priming vaccination using data from previous studies [4,14]. Table S2: Association between adverse events in the priming vaccination and “hesitancy” or “not sure” of the booster dose of vaccination. Table S3: The 7Cs of vaccination readiness (*n* = 6172). Table S4: Cumulative number of infections, deaths, and vaccinations as of the start date of the COVID-19 booster dose vaccination intention survey in each country. Figure S1: Comparison of 7C scale total scores according to vaccination intent.
