# Supplementary material for: Acceptance of Booster COVID-19 Vaccine and Its Association with Components of Vaccination Readiness in the General Population: A Cross-Sectional Survey for Starting Booster Dose in Japan
Source: Vaccines (Basel). 2022 Jul 8;10(7):1102. doi: 10.3390/vaccines10071102 (PMC9323594; doi:10.3390/vaccines10071102)
Supplement: Supplementary file 1 [file vaccines-10-01102-s001.zip › vaccines-1773746-supplementary.pdf]

Supplementary Table S1. Logistic regression analysis of factors associated with acceptance of the priming vaccination using data from previous studies [1, 2].

	**Crude OR**	**95% Cl**	***p*-value**	**Adjusted* OR**	**95% Cl**	***p*-value**	**Eta****
Sex	0.66	0.60—0.72	<0.001	0.67	0.60–0.75	<0.001	0.03
Age	1.12	1.09—1.16	<0.001	1.08	1.03–1.12	<0.001	
Child	1.36	1.24–1.49	<0.001	1.18	1.04–1.33	0.011	
Annual household income	0.74	0.66–0.83	<0.001	1.19	1.05–1.35	0.015	
Underlying disease	1.50	1.37—1.66	<0.001	0.86	0.76—0.97	<0.001	
Social norms	8.24	7.44—9.12	<0.001	8.02	7.13—9.03	<0.001	0.36
Confidence	2.46	2.30—2.62	<0.001	2.43	2.25—2.62	<0.001	0.14
Complacency	1.37	1.29—1.44	<0.001	1.41	1.32—1.51	<0.001	0.05
Constraints	1.62	1.55—1.70	<0.001	1.56	1.47—1.65	<0.001	0.08
Calculation	3.72	3.42—4.05	<0.001	3.58	3.24—3.95	<0.001	0.17
Collective responsibility	1.83	1.73—1.95	<0.001	1.84	1.71—1.97	<0.001	0.09

* Adjusted for sex, age, children, underlying disease status, and annual household income. However, sex, age, children, underlying disease status, and household income were adjusted all at once.

**The effect size (eta squared) was calculated with the General Linear Model, adjusted for age, children, underlying disease status, and annual household income.

OR: odds ratio; CI: confidence interval

COVID-19 vaccine acceptance was defined as follows: in the January survey, “If the COVID-19 vaccine were approved, would you want to be vaccinated?”, to which respondents answered on a 5-point Likert scale of “strongly disagree”, “disagree”, “neither agree nor disagree”, “agree”, and “strongly agree”. We defined “strongly agree” and “agree” as “acceptance.” The percentage of “acceptance” was 48.3% (n = 3480).

For each of these items, the following questions were asked, and the answers were obtained on a 5-point Likert-type rating scale ranging from “strongly disagree” to “strongly agree”, as described.
The scoring for “Constraints” is the reverse of other items. Cronbach's alpha for these six items was 0.66.

Social norms: If most people take a booster dose, I will do, too.
Confidence: Vaccines are safe.
Complacency: I am worried about getting COVID-19.
Constraints: I have difficulty getting immunized (no time, far medical institutions, etc.)
Calculation: Vaccines are effective.
Collective responsibility: My vaccination is important for the health of others in my community.

Supplementary Table S2. Association between adverse events in the priming vaccination and "hesitancy" or "not sure" of the booster dose of vaccination.

	**Crude OR**	**95% Cl**	***p*-value**	**Adjusted* OR**	**95% Cl**	***p*-value**	**Eta****
Lumps at the vaccination site	0.86	0.73–1.01	0.073	1.09	0.90—1.32	0.391	0.06
Itching at the vaccination site	1.24	1.04—1.46	0.015	1.12	0.91—1.38	0.303	0.06
Pain at the vaccination site	1.06	0.93—1.20	0.389	0.88	0.76—1.03	0.116	0.06
Redness at the vaccination site	1.03	0.88—1.19	0.740	0.96	0.80–1.15	0.650	0.06
Swelling of the inoculation site	1.11	0.98–1.26	0.116	0.96	0.82–1.12	0.579	0.06
Fever	1.73	1.53 -1.95	<0.001	1.28	1.10–1.49	0.002	0.06
Tiredness, fatigue	1.62	1.43 -1.82	<0.001	1.21	1.04–1.40	0.015	0.06
Headache	1.85	1.62–2.12	<0.001	1.28	1.08–1.51	0.004	0.06
Chills	1.98	1.68–2.32	<0.001	1.46	1.20–1.78	0.000	0.06
Vomiting	2.35	1.58–3.50	<0.001	1.80	1.14–2.86	0.012	0.06
Diarrhea	1.97	1.35–2.87	<0.001	1.60	1.00–2.54	0.049	0.06
Muscular pain	1.54	1.34–1.76	<0.001	1.28	1.09–1.51	0.003	0.06
Arthralgia	2.07	1.75–2.46	<0.001	1.64	1.33–2.03	<0.001	0.07
Anaphylactic shock	2.64	1.06–6.57	0.037	2.58	0.82–8.10	0.106	0.06
None	0.82	0.66–1.03	0.082	1.09	0.83–1.43	0.529	0.06

* Adjusted for sex, age, children, underlying disease status, and household income.

**The effect size (eta squared) was calculated using the General Linear Model, adjusted for age, children, underlying disease status, and household income.

Supplementary Table S3. The 7Cs of vaccination readiness (n = 6172)

	n	(%)
**I am convinced the appropriate authorities do only allow effective and safe vaccines. (Confidence)**		
Strongly disagree	137	2.2
Almost disagree	178	2.9
Probably disagree	761	12.3
Neither or not	1969	31.9
Probably agree	1889	30.6
Almost agree	1009	16.4
Strongly agree	229	3.7
**I get vaccinated because it is too risky to get infected. (Complacency)**		
Strongly disagree	38	0.6
Almost disagree	60	1.0
Probably disagree	227	3.7
Neither or not	894	14.5
Probably agree	1684	27.3
Almost agree	2072	33.6
Strongly agree	1197	19.4
**Vaccinations are so important to me that I prioritize getting vaccinated over other things. (Constraints)**		
Strongly disagree	113	1.8
Almost disagree	170	2.8
Probably disagree	665	10.8
Neither or not	1892	30.7
Probably agree	1941	31.5
Almost agree	1065	17.3
Strongly agree	326	5.3
**I only get vaccinated when the benefits outweigh the risks. (Calculation)**		
Strongly disagree	228	3.7
Almost disagree	793	12.9
Probably disagree	1495	24.2
Neither or not	2014	32.6
Probably agree	1020	16.5
Almost agree	380	6.2
Strongly agree	242	3.9
**I see vaccination as a collective task against the spread of diseases. (Collective responsibility)**		
Strongly disagree	41	0.7
Almost disagree	85	1.4
Probably disagree	214	3.5
Neither or not	893	14.5
Probably agree	2122	34.4
Almost agree	2091	33.9
Strongly agree	726	11.8
**It should be possible to sanction people who do not follow the vaccination recommendations by health authorities. (Compliance)**		
Strongly disagree	806	13.1
Almost disagree	745	12.1
Probably disagree	1679	27.2
Neither or not	1814	29.4
Probably agree	788	12.8
Almost agree	208	3.4
Strongly agree	132	2.1
**Vaccinations cause diseases and allergies that are more serious than the diseases they ought to protect from. (Conspiracy)**		
Strongly disagree	50	0.8
Almost disagree	160	2.6
Probably disagree	827	13.4
Neither or not	2346	38.0
Probably agree	1754	28.4
Almost agree	778	12.6
Strongly agree	257	4.2

Supplementary Table S4. Cumulative number of infections, deaths, and vaccinations as of the start date of the COVID-19 booster dose vaccination intention survey in each country.

	Date of survey	Start date of a booster dose	The intention of booster dose (%)	COVID-19 infection status of study subjects (%)	Cumulative number of infections per million people on the date of survey start [3]	Cumulative number of deaths per million on the survey start date [3]	Percent of Vaccine 2 doses at the start date of the survey (%) [3]
America (Yadete et al.) [4]	14–19 July 2021	17 September 2021 (65 years of age and older)	79.1	-------------------	102311	1825	50.5
Italy (Folcarelli et. al) [5]	16 November–6 December 2021	1 December 2021 (18 years old and older)	85.7	9.6	80723	2201	73.4
Poland (Rzymski et. al) [6]	8–9 September 2021	2 November 2021 (18 years old and older)	71.0	21	76503	1994	50.1
Japan (This study)	20–22 December 2021	1 December 2021 (18 years old and older)	78.3	1	13717	145	78.2

Supplementary Figure S1. Comparison of 7C scale total scores by vaccination intent.

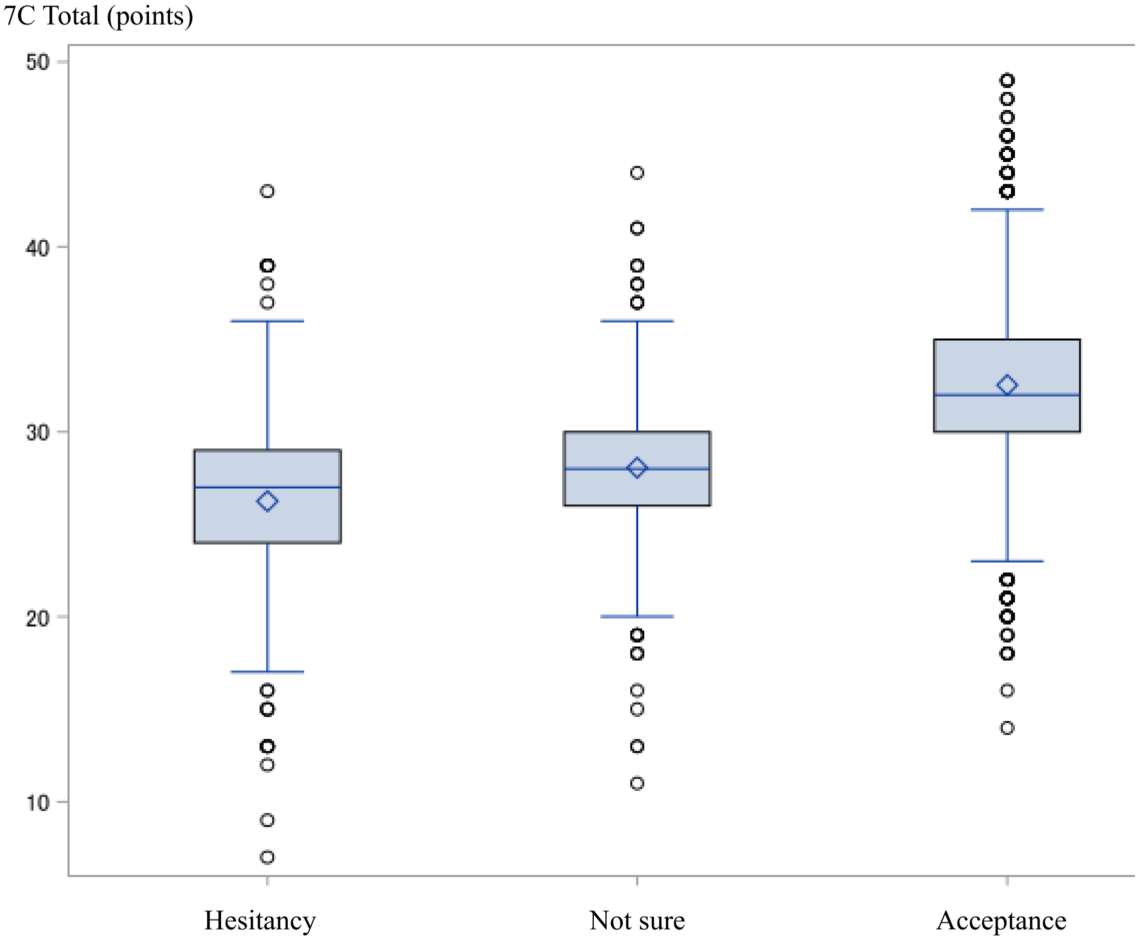

References

1. Hara, M., M. Ishibashi, A. Nakane, T. Nakano and Y. Hirota. "Differences in covid-19 vaccine acceptance, hesitancy, and confidence between healthcare workers and the general population in japan." 9 (2021): 1389.
2. Tokiya, M., M. Hara, A. Matsumoto, S. Mohanmad, Ashenagar, T. Nakano and Y. Hirota. "Association of vaccine confidence and hesitancy in three phases of covid-19 vaccine approval and introduction in japan." Vaccines **2020**, *10*, 423
3. Global Change Data Lab. "Our world in data." D. explorer. https://ourworldindata.org/coronavirus. march 09 2022. (accessed on 3 Feb 2022)
4. Yadete, T.; Batra, K.; Netski, D. M.; Antonio, S.; Patros, M. J.; Bester, J. C. Assessing acceptability of COVID-19 vaccine booster dose among adult Americans: a cross-sectional study. *Vaccines (Basel)* **2021**, *9,* 1424. 10.3390/vaccines9121424
5. Folcarelli, L.; Miraglia del Giudice, G.; Corea F.; Angelillo, I. F. Intention to receive the COVID-19 vaccine booster dose in a university community in Italy. *Vaccines (Basel)* **2022**, *10,* 146. 10.3390/vaccines10020146
6. Rzymski, P.; Poniedziałek, B.; Fal, A. Willingness to receive the booster COVID-19 vaccine dose in Poland. *Vaccines (Basel)* **2021**, *9,* 1286. 10.3390/vaccines9111286
